# Effect of Silodosin, an Alpha1A-Adrenoceptor Antagonist, on Ventral Prostatic Hyperplasia in the Spontaneously Hypertensive Rat

**DOI:** 10.1371/journal.pone.0133798

**Published:** 2015-08-26

**Authors:** Shogo Shimizu, Takahiro Shimizu, Panagiota Tsounapi, Youichirou Higashi, Darryl T. Martin, Kumiko Nakamura, Masashi Honda, Keiji Inoue, Motoaki Saito

**Affiliations:** 1 Department of Pharmacology, Kochi Medical School, Kochi University, Nankoku, Japan; 2 Department of Surgery, Division of Urology, Tottori University School of Medicine, Yonago, Japan; 3 Department of Urology, Yale University School of Medicine, New Haven, CT, United States of America; 4 Department of Urology, Kochi Medical School, Kochi University, Nankoku, Japan; King's College London, UNITED KINGDOM

## Abstract

**Background:**

A decreased prostatic blood flow could be one of the risk factors for benign prostatic hyperplasia/benign prostatic enlargement. The spontaneously hypertensive rat (SHR) shows a chronic prostatic ischemia and hyperplastic morphological abnormalities in the ventral prostate. The effect of silodosin, a selective alpha_1A-_adrenoceptor antagonist, was investigated in the SHR prostate as a prostatic hyperplasia model focusing on prostatic blood flow.

**Methods:**

Twelve-week-old male SHRs were administered perorally with silodosin (100 μg/kg/day) or vehicle once daily for 6 weeks. Wistar Kyoto (WKY) rats were used as normotensive controls and were treated with the vehicle. The effect of silodosin on blood pressure and prostatic blood flow were estimated and then the prostates were removed and weighed. The tissue levels of malondialdehyde (MDA), interleukin-6 (IL-6), chemokine (C-X-C motif) ligand 1/cytokine-induced neutrophil chemoattractant 1 (CXCL1/CINC1), tumor necrosis factor-alpha (TNF-α), transforming growth factor beta 1 (TGF-β1), basic fibroblast growth factor (bFGF) and alpha-smooth muscle actin (α-SMA) were measured. The histological evaluation was also performed by hematoxylin and eosin staining.

**Results:**

There was a significant increase in blood pressure, prostate weight, prostate body weight ratio (PBR), tissue levels of MDA, IL-6, CXCL1/CINC1, TNF-α, TGF-β1, bFGF and α-SMA in the SHR compared to the WKY rat. The ventral prostate in the SHR showed the morphological abnormalities compared to the WKY rat. Prostatic blood flow was decreased in the SHR. However, treatment with silodosin significantly restored the decreased prostatic blood flow in the SHR. Moreover, silodosin normalized tissue levels of MDA, IL-6, CXCL1/CINC1, TNF-α, TGF-β1, bFGF and α-SMA, and it ameliorated ventral prostatic hyperplasia in the SHR excluding blood pressure. Silodosin decreased PBR but not prostate weight in the SHR.

**Conclusions:**

Silodosin can inhibit the progression of prostatic hyperplasia through a recovery of prostatic blood flow.

## Introduction

Benign prostatic hyperplasia (BPH) is one of the most common diseases among elderly men. Epidemiological data show that approximately 50% of patients develop lower urinary tract symptoms (LUTS) due to BPH/benign prostatic enlargement (BPE) [1]. BPH/BPE consist of overgrowth of epithelial and stromal cells within the transitional zone and periurethral area [2]. Although aging and androgens are two established risk factors for the development of BPH/BPE, serum androgen levels are generally decreased in elderly males and are not correlated with prostate volume [2]. There is some evidence that vascular risk factors may be associated with progression of BPH/BPE [3,4]. Clinical data showed that BPH and hypertension occur with increasing prevalence with older age, and are more likely to be pathophysiologically relevant [5,6]. De Nuzio et al. suggested that BPH related LUTS are caused by multiple factors including pelvic atherosclerosis followed by chronic ischemia of prostate, and chronic inflammation in the prostate [7]. Inflammation with cytokines and macrophage infiltration might induce proliferation of prostatic tissue [3,8]. Recent basic and clinical research suggest that prostatic inflammation could be one of the central mechanisms in the development of BPH/BPE [9,10]. In addition, chronic histological inflammation was found in more than 90% of specimens obtained from transurethral resection of the prostate [11].

The spontaneously hypertensive rat (SHR) is commonly used as a genetically hypertensive rat model of BPH/BPE [12]. The SHR exhibits decreased prostatic blood flow and develops hyperplastic morphological abnormalities in the ventral prostate as early as 15 weeks [13,14]. However, our previous data showed that there was no statistical difference in dihydrotestosterone (androgen hormone) levels in the prostate between the SHR and the WKY rat, which is used as a normotensive control model [15]. These reports indicate that the ventral prostate of the SHR can be a good model for human BPH/BPE. Our previous report showed chronic treatment with nicorandil, a vasodilator, significantly increased prostatic blood flow and inhibited the progression of ventral prostatic hyperplasia in the SHR [15]. A recent study showed that chronic pelvic ischemia induced distinct functional and morphological changes in the rat ventral prostate. Pretreatment with tadalafil, a phosphodiesterase type 5 inhibitor, has been shown to decrease collagen deposition in the ventral prostate caused by arterial endothelial injury [16]. Thus, blood flow in the prostate plays an important role in prostatic hyperplasia development.

Alpha (α)_1_-adrenoceptor antagonists are the most frequently used drugs for treatment of BPH related LUTS. It is widely known that α_1_-adrenoceptor antagonists relax prostate smooth muscle and decrease urethral resistance, thereby alleviating LUTS [17]. Pinggera et al. demonstrated that α_1_-adrenoceptor antagonists ameliorated chronic ischemia of the lower urinary tract in patients with LUTS [18]. Doxazosin, an α_1_-adrenoceptor antagonist, restored prostatic blood flow in the SHR [14]. Additionally, chronic treatment with terazosin, an α_1_-adrenoceptor antagonist, inhibited hyperplastic changes in the SHR prostate [19]. Thus, we investigated whether silodosin, a selective α_1A_-adrenoceptor antagonist, could ameliorate blood flow, inflammation and hyperplasia in the SHR ventral prostate.

## Material and Methods

### Animal preparation

The layout of the experiments were approved by the Institutional Animal Care and Use committee of Kochi University (Permit Number: H-113), which were in accordance with the guidelines for proper conduct of animal experiments from the Science Council of Japan. The protocol was approved by the committee on Ethics of Animal Experiments of the Kochi University (Permit Number: H-113). All studies involving animals were reported in accordance with ARRIVE guidelines [20]. In this study, we used 12 weeks old male SHRs and WKY rats. The animals were purchased from SLC Japan (Hamamatsu, Japan). All animals were kept under identical conditions of temperature and humidity, and had access to food and drinking water *ad libitum*. When the animals (n = 24) reached 12 weeks of age, they were randomly divided into three groups (n = 8/group): an age-matched WKY rat group treated with vehicle (0.5% methylcellulose in distilled water) perorally (p.o.) (WKY), SHRs treated with vehicle p.o. (SHR), and SHRs treated with silodosin at a daily dose of 100 μg/kg, p.o. (SHR+Sil100). Taking into consideration that the US Food and Drug Administration suggested an oral dose of silodosin to be taken at 8 mg once daily, we adopted the dosage of 100 μg/kg, which was administered (p.o.) once daily for 6 weeks in accordance with our previous experiments [21]. Also, our preliminary data showed no significant differences in prostatic blood flow or basic fibroblast growth factor (bFGF) in the prostate between the SHRs treated with two different doses of silodosin (i.e. 100 or 300 μg/kg p.o.) once daily for six weeks. Thus, in subsequent experiments we decided to use only a dose of 100 μg/kg p.o.. After 6 weeks of treatment, when the animals reached 18 weeks of age, blood pressure and heart rate were measured with the tail cuff method, which included warming the whole animal body in the absence of anesthesia (BP-98A-L, Softron, Tokyo, Japan) [15]. Subsequently, prostatic blood flow was measured under intraperitoneally (i.p.) administered sodium pentobarbital anesthesia (50 mg/kg) as described below; afterwards, the rats were sacrificed with an overdose of sodium pentobarbital (60 mg/kg i.p.). The isolated prostates were frozen at -80°C until measurements of tissue were performed. The remaining parts of ventral and dorsolateral prostate were placed in 10% neutral buffered formalin for histopathological evaluation [15].

### Measurement of prostatic blood flow

Prostatic blood flow was measured using the hydrogen clearance method [15]. The hydrogen electrode (80 μm diameter, UHE-201C; Unique Medical Co., Tokyo, Japan) was inserted into the ventral prostate. A rod-type Ag/AgCl reference electrode (UHE-001; Unique Medical Co.) was inserted between the skin and musculature region in the abdomen.

### Measurement of oxidative stress in the prostate

The levels of malondialdehyde (MDA), a widely used marker of lipid peroxidation, in prostatic tissue homogenates were identified using a commercially available kit (NWLSSTM Malondialdehyde Assay; Northwest Life Science Specialties LLC., Vancouver, WA). The MDA concentrations were normalized by the protein content.

### Measurements of inflammatory cytokines in the prostate

Commercially available kits were used to identify homogenates levels of interleukin-6 (IL-6) (Quantikine ELISA Rat IL-6 Immunoassay, R6000B; R&D Systems Inc, MN), chemokine (C-X-C motif) ligand 1/cytokine-induced neutrophil chemoattractant 1 (CXCL1/CINC1) (Rat CXCL1/CINC1 Quantikine ELISA; R&D Systems Inc.) and tumor necrosis factor-α (TNF-α) (Rat TNF-α ELISA kit; RayBiotech, Inc, GA). Obtained markers were normalized by the protein content. CXCL1/CINC1 is the rat homologue to human IL-8.

### Transforming growth factor beta 1 (TGF-β1) and bFGF measurements in the prostate

For the measurement of TGF-β1 and bFGF, we used commercially available kits (Quantikine ELISA mouse/rat/porcine/canine TGF-β1 immunoassay MB100B; R&D Systems Inc.) (Quantikine ELISA mouse/rat FGF basic immunoassay MFB00; R&D Systems Inc). The concentrations of TGF-β1 and bFGF were normalized by the protein content.

### Protein assay in the prostatic homogenates

Protein concentration was determined using a commercially available kit (Protein Assay Rapid Kit; Wako Pure Chemical, Osaka, Japan). Bovine serum albumin was used as the standard.

### Western blot analysis of alpha-smooth muscle actin (α-SMA)

Western blot analysis was performed according to previous methods [15]. Protein samples (50 μg) were subjected to SDS-polyacrylamide gel electrophoresis on 12% gradient gels, and the separated proteins were transferred to polyvinylidene difluoride membranes. The membrane was blocked with 5% nonfat milk in Tris-Buffered Saline containing 0.1% Tween 20 (TBS-T), and then incubated overnight on a shaker at 4°C with antibodies for α-SMA (1:400), and anti-β-actin (1:1,000) in 5% nonfat milk in TBS-T. After washing with TBS-T (10 min×3 times), the membrane was incubated for 1 hour at room temperature on a shaker with a 1:3,000 dilution of horseradish peroxidase-conjugated rabbit antibody in 5% nonfat milk in TBS-T. After washing with TBS-T (10 min×3 times), the detection was performed using an enhanced chemiluminescence reagent (Millipore Corporation, Billerica, MA). The intensity of target bands was quantified by densitometry using Malti Gauge software (FUJIFILM, Tokyo, Japan). A rabbit polyclonal anti-α-SMA (ab5694, Abcam, Tokyo, Japan) and a rabbit polyclonal anti-β-actin (54590, AnaSpec, Inc., San Jose, CA) were used as primary antibodies. β-Actin was used as a control for normalization of α-SMA.

### Histological examinations

After fixation, the tissues were embedded in paraffin, and five-micron-thick tissue sections were cut from the paraffin blocks. All of the prostate specimens were stained using hematoxylin and eosin (HE). Each section was viewed under a light microscope at a magnification of ×40–400 and morphological changes were evaluated by two blinded investigators (S.S. and H.Y.). ImageJ 1.48 software (NIH, Bethesda, MD) was used to calculate the HE stained gland area in the rat ventral prostate. The stained area in the gland indicated the degree of proliferation of epithelial components. Under blind conditions, we randomly counted 10 glands in each section and then quantified the glandular epithelial area as stained area per glandular area under 100-fold magnification. The mean percent area density was also evaluated for each group.

### Data analysis

Quantitative data are presented as means ± SEM and were compared among multiple experimental groups using analysis of variance and Fisher’s multiple comparison tests. *P-*values less than 0.05 were considered statistically significant.

### Drugs and chemicals

Silodosin was supplied by the Daiichi-Sankyo Pharmaceutical Co. Ltd (Tokyo, Japan). All other chemicals were commercially available and of reagent grade quality. 

## Results

### General features of the animals

After six weeks of silodosin or vehicle treatment, body weights in the SHR and SHR+Sil100 groups were significantly lower compared to the WKY group (Table 1). The prostate weight and the prostate body weight ratio (PBR) in the SHR and SHR+Sil100 groups were significantly higher than those in the WKY group. Treatment with silodosin significantly decreased PBR, but not prostate weight in the SHR. Heart rate was significantly lower in the SHR group compared to the WKY group. Heart rate in the SHR+Sil100 group was not significantly different from either the WKY group or the SHR group. The SHR+Sil100 group demonstrated significantly higher mean blood pressure compared to the WKY group, while there were no significant differences when compared to the SHR group (Table 1).

**Table 1:** The General Features.

	Groups		
Factors	WKY	SHR	SHR+Sil100
**Body Weight (g)**	**402 ± 5**	**326 ± 16** †	**336 ± 12** †
**Prostate Weight (mg)**	**630 ± 35**	**926 ± 31** †	**863 ± 54** †
**PBR (×10** ^**−3**^ **)**	**1.57 ± 0.09**	**2.86 ± 0.07** †	**2.56 ± 0.11** † #
**Heart Rate (bpm)**	**338.0 ± 9.6**	**302.4 ± 3.9** †	**324.0 ± 9.8**
**Mean Blood Pressure (mmHg)**	**106.5 ± 1.1**	**166.0 ± 5.9** †	**154.7 ± 4.5** †

PBR: Prostate body weight ratio (prostate weight/body weight); WKY: 18-week-old WKY rats treated with vehicle, p.o.; SHR: 18-week-old SHRs treated with the vehicle, p.o.; SHR+Sil100: 18-week-old SHRs treated with silodosin at a daily dose of 100 μg/kg, p.o.

Data are shown as mean ± SEM of eight separate determinations in each group.

^†^: Significantly different from the WKY group (*P* < 0.05);

^#^: Significantly different from the SHR group (*P* < 0.05).

### Prostatic blood flow

**Fig 1:**
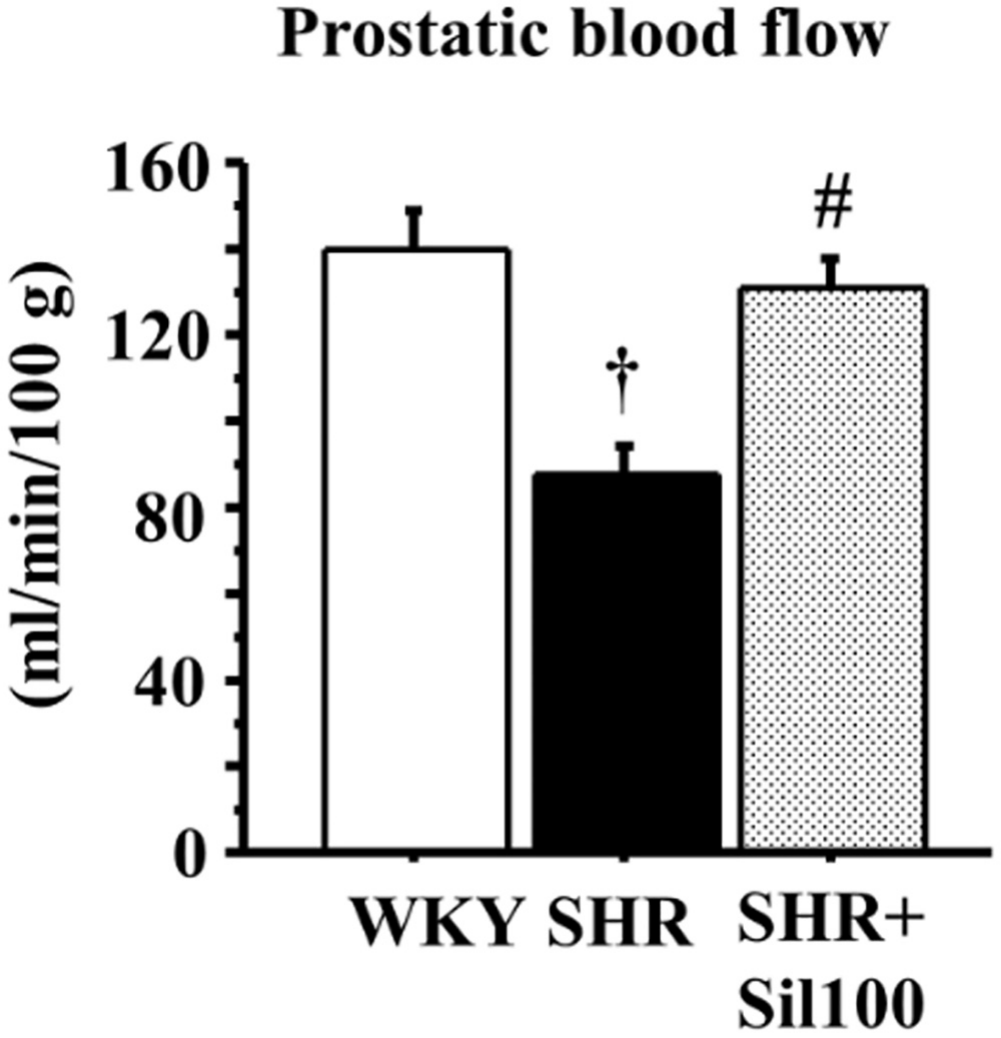
Blood flow in the ventral prostate. The effect of silodosin on blood flow in the ventral prostate of the 18-week-old Wistar-Kyoto rat group (WKY), the 18-week-old SHR group (SHR) and the 18-week-old SHRs treated with silodosin at a daily dose of 100 μg/kg, p.o. (SHR+Sil100). Data are shown as mean ± SEM of eight separate determinations in each group. †: Significantly different with the WKY group; #: Significantly different with the SHR group (*P* < 0.05 is a level of significance).

Prostatic blood flow in the SHR group was significantly lower compared to the WKY group. Treatment with silodosin significantly increased prostatic blood flow compared to the SHR group (Fig 1).

### MDA concentrations in the ventral prostate

**Fig 2:**
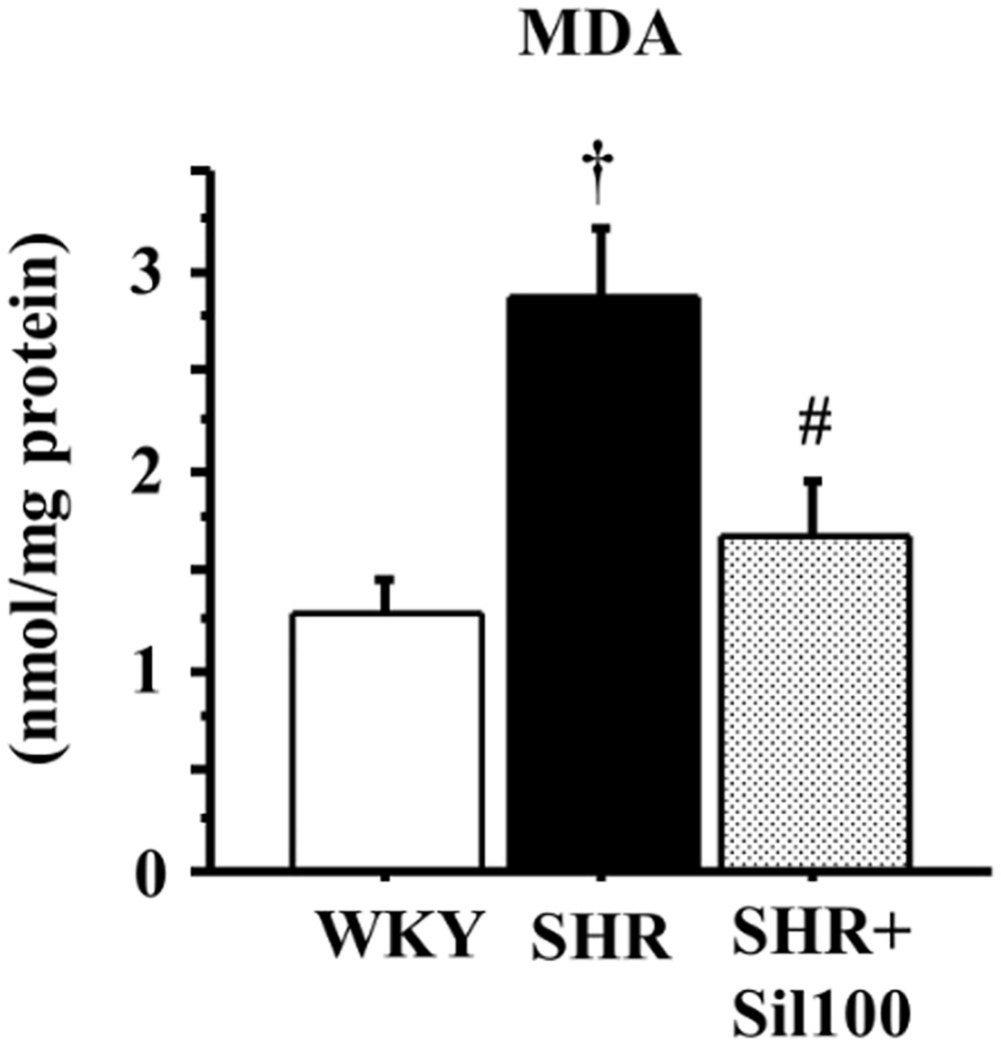
MDA levels in the ventral prostate. The effect of silodosin on MDA levels in the ventral prostate of the 18-week-old Wistar-Kyoto rat group (WKY), the 18-week-old SHR group (SHR) and the 18-week-old SHRs treated with silodosin at a daily dose of 100 μg/kg, p.o. (SHR+Sil100). Data are shown as mean ± SEM of eight separate determinations in each group. †: Significantly different with the WKY group; #: Significantly different with the SHR group (*P* < 0.05 is a level of significance).

Oxidative stress levels were significantly increased in the SHR ventral prostates compared to the WKY group, as evaluated by MDA concentrations in the prostate tissues. Treatment with silodosin significantly decreased MDA concentrations compared to the SHR group. There were no statistically significant differences in MDA concentrations between the WKY and SHR+Sil100 groups (Fig 2).

### IL-6, CXCL1/CINC1 and TNF-α levels in the ventral prostate

**Fig 3:**
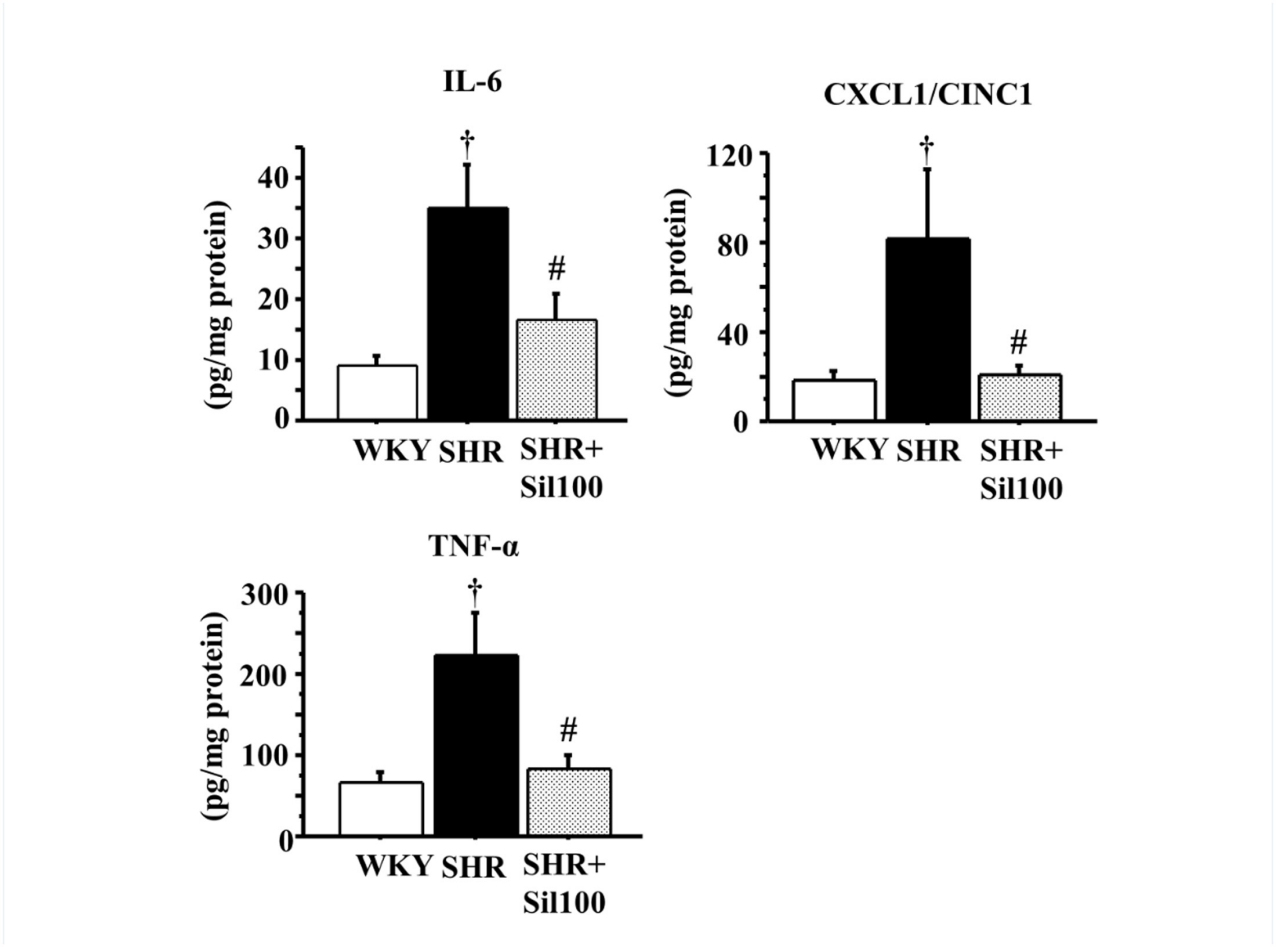
IL-6, CXCL1/CINC1 and TNF-α levels in the ventral prostate. Tissue levels of IL-6 (left upper panel), CXCL1/CINC1 (right upper panel), and TNF-α (left lower panel) in the ventral prostate are shown. WKY: 18-week-old Wistar-Kyoto rat group; SHR: 18-week-old SHR group; SHR+Sil100: 18-week-old SHRs treated with silodosin at a daily dose of 100 μg/kg, p.o.. Data are shown as mean ± SEM of eight separate determinations in each group. †: Significantly different with the WKY group; #: Significantly different with the SHR group (*P* < 0.05 is a level of significance).

Our results revealed a significant increase in IL-6, CXCL1/CINC1 and TNF-α levels in the ventral prostate of the SHR group compared to the WKY group. Treatment with silodosin significantly decreased levels of IL-6, CXCL1/CINC1 and TNF-α in the SHR ventral prostate compared to the vehicle control SHR ventral prostate. There were no statistically significant differences between the WKY group and the SHR+Sil100 group with respect to IL-6, CXCL1/CINC1 and TNF-α levels (Fig 3).

### TGF-β1, bFGF and α-SMA levels in the ventral prostate

**Fig 4:**
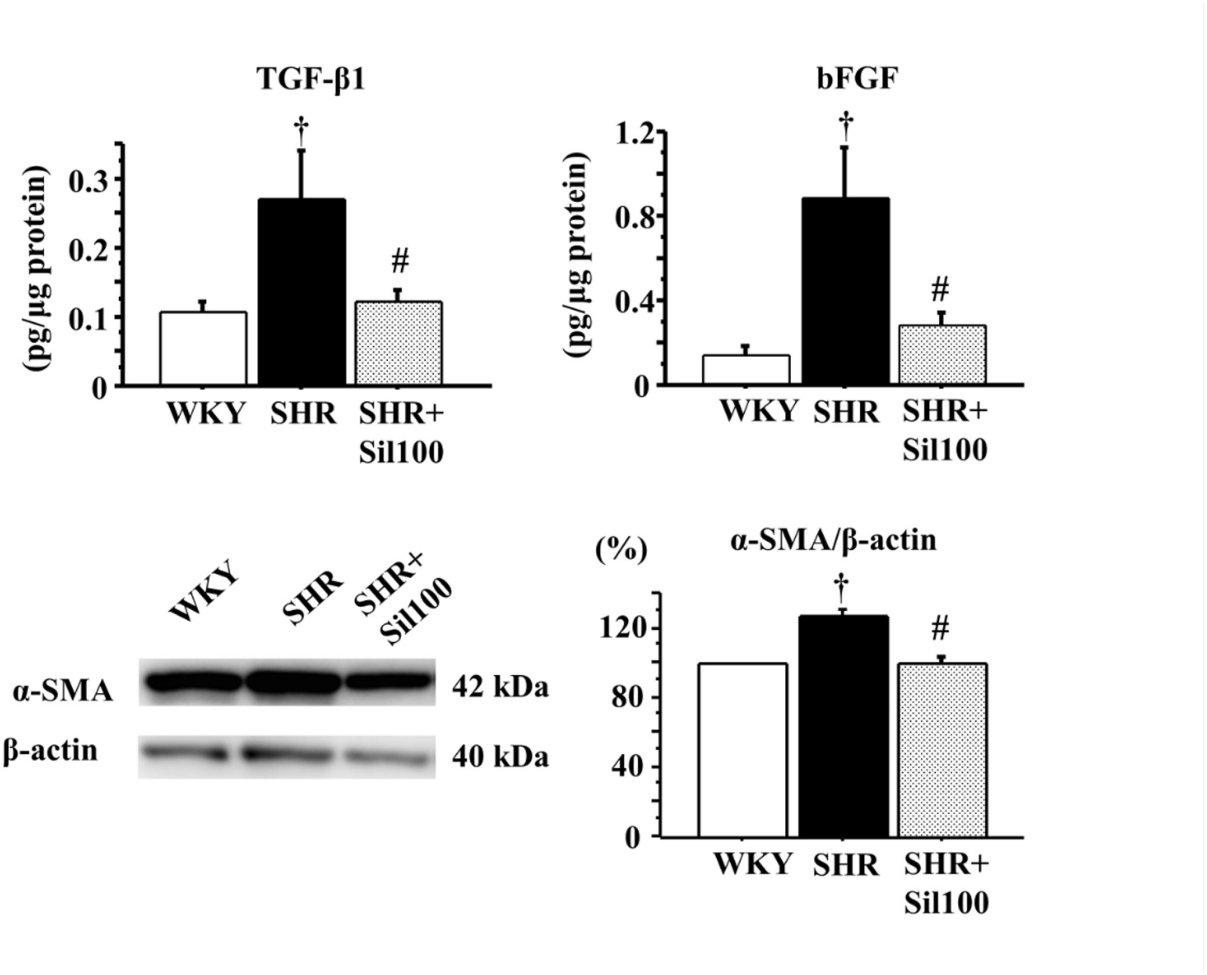
TGF-β1, bFGF and α-SMA levels in the ventral prostate. Tissue levels of TGF-β1 (left upper panel), bFGF (right upper panel), α-SMA (right lower panel), and a representative blot of α-SMA in the ventral prostate (left lower panel) are shown. WKY: 18-week-old Wistar-Kyoto rat group; SHR: 18-week-old SHR group; SHR+Sil100: 18-week-old SHRs treated with silodosin at a daily dose of 100 μg/kg, p.o.. Data are shown as mean ± SEM of eight separate determinations in each group. †: Significantly different with the WKY group; #: Significantly different with the SHR group (*P* < 0.05 is a level of significance).

There was a significant increase in TGF-β1, bFGF and α-SMA levels in the SHR group compared to the WKY group. Treatment with silodosin significantly decreased TGF-β1 and bFGF levels, and α-SMA protein expression compared to the SHR group. Also, there were no statistically significant differences between the WKY group and the SHR+Sil100 group with respect to TGF-β1, bFGF and α-SMA levels (Fig 4).

### Histological evaluation of the prostatic tissues

**Fig 5:**
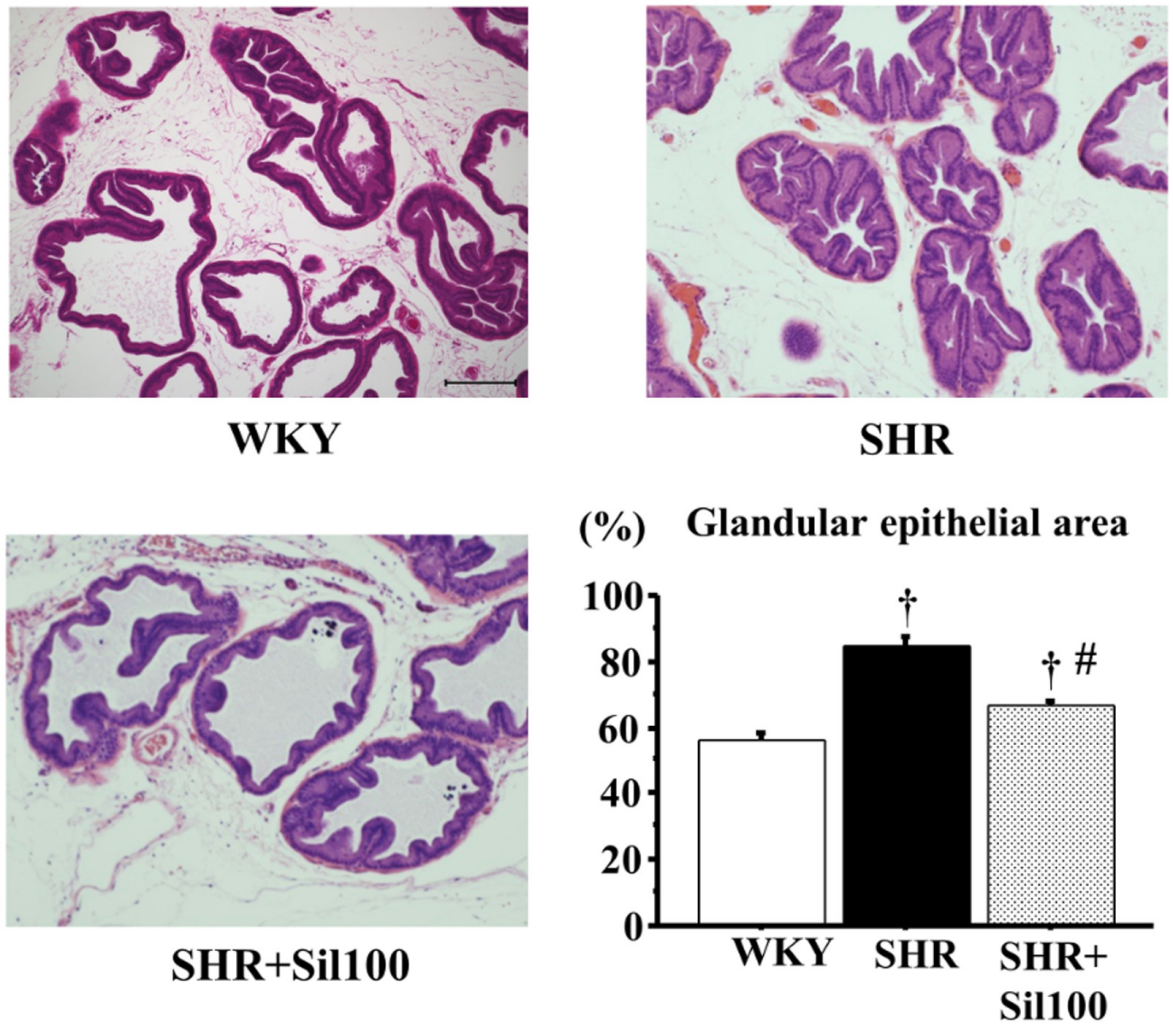
Histological changes in the ventral prostate of the SHR animals. The ventral prostate in the WKY group (left upper panel), the SHR group (right upper panel), and the SHR+Sil100 group (left lower panel) are shown. Glandular epithelial area (%) (HE stained area/ventral prostate glandular area×100) are depicted in the right lower panel. WKY: 18-week-old Wistar-Kyoto rat group; SHR: 18-week-old SHR group; SHR+Sil100; 18-week-old SHRs treated with silodosin at a daily dose of 100 μg/kg, p.o.. Original magnification: ×100. The scale bar is 200 μm. †: Significantly different with the WKY group; #: Significantly different with the SHR group (*P* < 0.05 is a level of significance).

In contrast to histological sections in the WKY group, ventral prostate in the SHR group showed morphological abnormalities, characterized by increased branching of epithelial cells. Treatment with silodosin decreased these morphological alterations observed in the SHR group (Fig 5). For calculations using ImageJ, glandular epithelial area (stained area/glandular area) in the SHR group and the SHR+Sil100 group were significantly higher compared to the WKY group, indicating hyperplasia of the prostate. As well, the glandular epithelial area in the SHR+Sil100 group was significantly lower compared to the SHR group (Fig 5).

## Discussion

The present study showed that chronic treatment with silodosin significantly ameliorated decreased prostatic blood flow, and increased oxidative stress, inflammatory cytokines, growth factors and morphological abnormalities in the SHR ventral prostate. On the other hand, silodosin failed to decrease blood pressure in the SHR. The management of prostatic blood flow recovery is a very important factor to ameliorate prostatic hyperplasia [15,16,22]. Pinggera et al. demonstrated that chronic ischemia of the prostate and bladder could be associated with LUTS [18]. Silodosin has a very higher selectivity for the α_1A_ subtype than for α_1B_ and α_1D_-adrenoceptor subtypes compared to other α_1_-adrenoceptor antagonists, while minimizing the undesirable effects on blood pressure [23]. Some reports demonstrated that chronic treatment with a selective α_1A_-adrenoceptor antagonist silodosin increased bladder blood flow in the SHR and in a rat model of chronic bladder ischemia induced by atherosclerosis. Also, the treatment subsequently improved bladder dysfunction [21,24]. α_1A_-Adrenoceptors are abundant in smooth muscle of the prostate [23]. Molecular analysis in human has shown 70% of the α_1_-adrenoceptor mRNA in the prostate was compromised of the α_1A_ subtype [25]. The mechanism for improvement of blood flow to the prostate with silodosin could be due to the blockade of α_1A_ subtype in the smooth muscle or microvessels (vascular smooth muscle) supplying blood flow to the prostate.

In the SHR, it is possible that ischemia or local hypoxia induces morphological structural alteration contributing to pathogenesis of BPH. The SHR prostate exhibited a high density of hypoxic cells that were localized in the epithelium when compared to the WKY rat [26]. Local hypoxia induces mild levels of reactive oxygen species (ROS), which can promote neovascularization and fibroblast-to-myofibroblast transdifferentiation and growth factors release (IL-8, TGF-β, FGF-7 and FGF-2) [3]. Under hypoxic conditions, growth factors stimulate not only inflammatory cells, but also epithelial and stromal cells, leading to prostatic enlargement [3]. Moreover, ROS produced by inflammatory cells create a positive feedback loop, which can amplify inflammation in human cells from BPH tissue [27]. Prostatic ischemia leads to generation of ROS and subsequent oxidative stress [15]. Oxidative stress has been considered to play a role in the development of BPH [15]. The current study shows that SHR has a significant increase in the concentration of MDA, an oxidative stress marker, in the prostate as compared to the WKY rat prostate. Prostatic hyperplasia in the SHR could be related to oxidative stress caused by lower prostatic blood flow. Treatment with silodosin significantly ameliorates increased MDA concentrations in the SHR ventral prostate. A previous study showed that silodosin could improve bladder blood flow, and ameliorated detrusor overactivity by reducing ROS produced by ischemia/reperfusion in atherosclerosis induced chronic bladder model [24]. Those data suggested that silodosin reduced oxidative stress via the recovery of prostatic blood flow in the SHR.

Inflammation of BPH is largely characterized by infiltration of activated lymphocytes and macrophages in the prostate. Inflammatory cytokines secreted from inflammatory cells may induce proliferation of both epithelial and stromal compartments, which results in prostate volume enlargement and BPH [28]. IL-6 and IL-8 are recognized as potent growth factors for prostatic epithelial and stromal cells [3]. TNF-α is a multifunctional cytokine that is thought to induce inflammation [3]. The current study demonstrates that the SHR significantly increased levels of IL-6, CXCL1/CINC1 and TNF-α in the prostate. Epidemiological studies also showed an elevation of IL-6, IL-8 and TNF-α in men with BPH as compared to normal prostate without BPH [9]. To our knowledge, this is the first study to report an increase in inflammatory cytokines in the SHR prostate. Moreover, chronic treatment with silodosin decreased these inflammatory cytokines in the SHR prostate. From our international literature searches, there were no pharmacological or biochemical evidence that silodosin had an anti-oxidative or an anti-inflammatory effect. A possible mechanism as to why silodosin ameliorated oxidative stress and inflammation in the prostate of SHR may be due to the improvement in the prostatic blood flow.

A variety of hormonal and paracrine factors (i.e. growth factors) stimulate prostatic growth and development. TGF-β1 and bFGF play critical roles in the regulation of prostatic growth and proliferation of stromal cells [29,30]. In the human prostate, TGF-β1 and bFGF are produced by stromal smooth muscle cells and are also secreted by epithelial cells of glands [30]. In the current study, silodosin significantly decreased levels of TGF-β1 and bFGF in the SHR ventral prostate compared to the vehicle control SHR ventral prostate. The α-SMA is recognized as a marker for prostatic fibroblast within prostatic stroma [16,31]. Previous reports demonstrated that α-SMA was abundant in stromal smooth muscle in BPH human prostate [32]. The current study showed that chronic treatment with silodosin decreased protein levels of α-SMA in the SHR ventral prostate compared to the vehicle control SHR ventral prostate. These data suggest that silodosin could inhibit the extent of hyperplasia in stromal tissue in the SHR.

Involvement of α_1_-adrenoceptors in prostatic growth and hyperplasia has been repeatedly suggested [32–34], even though the molecular mechanism is not well understood. Silodosin could inhibit growth factors via an increase in prostatic blood flow, and decrease in oxidative stress and inflammatory cytokines in the SHR ventral prostate. This study showed that epithelial cells of the SHR ventral prostate were taller in shape compared to the WKY. We evaluated the HE stained area in each ventral prostate gland to quantify the degree of proliferation of epithelial components. Treatment with silodosin decreased glandular epithelial area in the SHR compared to the vehicle control, suggesting that silodosin could inhibit the progression of prostatic hyperplasia. Also, silodosin decreased PBR, but not prostate weight, in the SHR. One limitation of this study is that the histological changes in the ventral prostate of 18-week-old SHRs are characterized as mainly epithelial (glandular) hyperplasia, which is considerably different from those of human BPH (fibromyo-glandular hyperplasia). While both epithelial and stromal cells contribute to human BPH/BPE, stromal cells play a bigger role. The previous experimental studies showed that treatment with other α_1_-adrenoceptor antagonists (i.e. terazosin and doxazosin) caused stromal regression and reduced growth in patients with BPH [32–34]. Thus, chronic treatment with silodosin could inhibit prostatic stromal growth in animal models as well as human BPH. However, in clinical studies, treatment with some α_1_-adrenoceptor antagonists (i.e. tamsulosin, alfuzosin and doxazosin) failed to reduce prostate volume, although chronic treatment with terazosin, α_1_-adrenoceptor antagonist was reported to reduce prostate volume [35–38]. Further evidence is needed to explain the discrepancy between the experimental and clinical data.

## Conclusions

Chronic treatment with silodosin has the potential to ameliorate prostatic blood flow, oxidative stress, inflammatory responses and growth factors in the SHR ventral prostate. Silodosin can inhibit the progression of prostatic hyperplasia through a recovery of prostatic blood flow.
